# Mild hypothermia after near drowning in twin toddlers

**DOI:** 10.1186/cc2926

**Published:** 2004-09-02

**Authors:** Ortrud Vargas Hein, Andreas Triltsch, Christoph von Buch, Wolfgang J Kox, Claudia Spies

**Affiliations:** 1 Department of Anesthesiology and Intensive Care Medicine, Charité, Campus Mitte, Humboldt University, Berlin, Germany; 2 Department of Anesthesiology and Intensive Care Medicine, Benjamin Franklin Medical Center, Free University, Berlin, Germany; 3 University Department of Pediatrics, University of Heidelberg, Mannheim, Germany

**Keywords:** children, mild hypothermia, near drowning, twins

## Abstract

**Introduction:**

We report a case of twin toddlers who both suffered near drowning but with different post-trauma treatment and course, and different neurological outcomes.

**Methods and results:**

Two twin toddlers (a boy and girl, aged 2 years and 3 months) suffered hypothermic near drowning with protracted cardiac arrest and aspiration. The girl was treated with mild hypothermia for 72 hours and developed acute respiratory dysfunction syndrome and sepsis. She recovered without neurological deficit. The boy's treatment was conducted under normothermia without further complications. He developed an apallic syndrome.

**Conclusion:**

Although the twin toddlers experienced the same near drowning accident together, the outcomes with respect to neurological status and postinjury complications were completely different. One of the factors that possibly influenced the different postinjury course might have been prolonged mild hypothermia.

## Introduction

Of drowning and near drowning victims who are younger than 20 years, 63–68% are 0–5 years old [1,2]. Of submersion events in the age group 1–4 years, 56% occurred in artificial pools [3]. Death from drowning is the second leading cause of accidental death in children [4], and one-third of all survivors have neurological damage [4]. Hypothermia frequently accompanies submersion accidents, especially in children with a relatively large ratio of surface area to body mass [3]. Mild hypothermia (32–34°C) reduces oxygen consumption by 7% per 1°C decrease in temperature, and reduces cerebral blood flow and cerebral intracranial pressure [5-7]. Temperature under 28°C leads to cardiocirculatory depression and finally cardiac arrest [3]. Hypoxaemia and capillary leak develop due to apnoea, regardless of whether aspiration occurs [3]. The degree of cerebral protection that can be expected due to hypothermia depends, among other factors, on the amount of time that elapses before induction of mild hypothermia [1,3,6]. Induced mild hypothermia for cerebral protection after near drowning accidents has yielded controversial results in terms of mortality and neurological outcome [1,3,8]. However, induced mild hypothermia after cardiac arrest has led to improved neurological results, whereas life-threatening complications such as infections and resultant sepsis may counter these neurological benefits [9].

We report here a case of twins who both suffered near drowning, but with different post-trauma treatment and different neurological outcomes.

## Case report

The twins (a girl and boy, aged 2 years and 3 months old) were found lifeless by their father in the neighbours' garden pond. It was early spring, and the toddlers had been unattended for at least 10 min. Bystander cardiopulmonary resuscitation (CPR) was performed. The emergency doctor could not palpate any pulse, the children were hypothermic, and the pupils were dilated and pupil reflexes absent. Both children had aspirated. Under CPR the children exhibited pulseless bradycardia on the electrocardiogram.

### The girl

**Figure 1:**
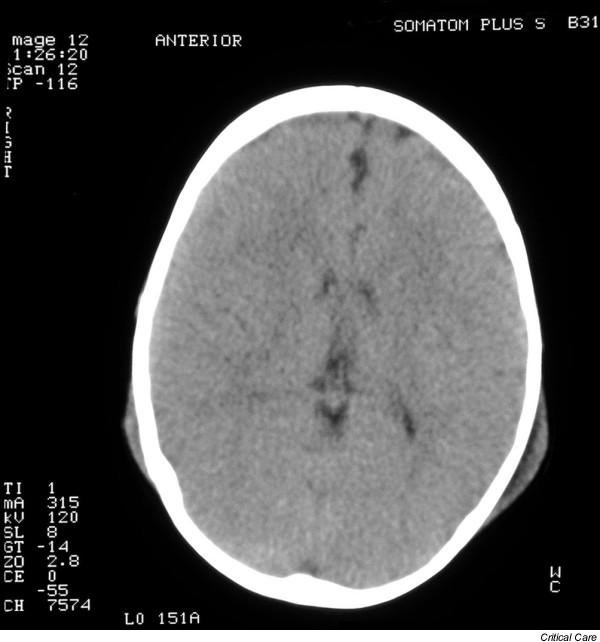
The girl: cranial computed tomography, done 7 hours after admission, showing cerebral oedema.

The girl was transported to a university hospital. Admission parameters are presented in Table 1. After rewarming to 32°C and successful CPR, 180 min after admission to the hospital, haemodynamic stability was achieved with adrenaline (epinephrine) infusion and the child was admitted to the intensive care unit (ICU). The pupils were slightly dilated with reaction to light and the corneal reflex was absent. Cranial computed tomography (CT; Fig. 1), done 7 hours after admission, revealed cerebral oedema; this was regressive, as indicated by cranial CT obtained 3 days later. Mannitol therapy and prolonged mild hypothermia (32–34°C) were begun the day of the accident. Repeated fundoscopy did not show signs of papillary congestion. Intracranial pressure was not monitored. Under sedation with fentanyl and midazolam to a Ramsay level of 6 and controlled mild hyperventilation (arterial CO_2 _tension 30–35 torr), mild hypothermia was continued and reduced gradually (0.5°C/8 hours). Seventy-two hours after the accident the child was normothermic without development of rebound hyperthermia. After rewarming the pupils were tight and reflexes present. Catecholamine therapy on admission to the ICU was switched to dobutamine and dopamine infusion. To achieve a mean arterial pressure greater than 70 mmHg, noradrenaline (norepinephrine) infusion had to be added.

**Figure 2:**
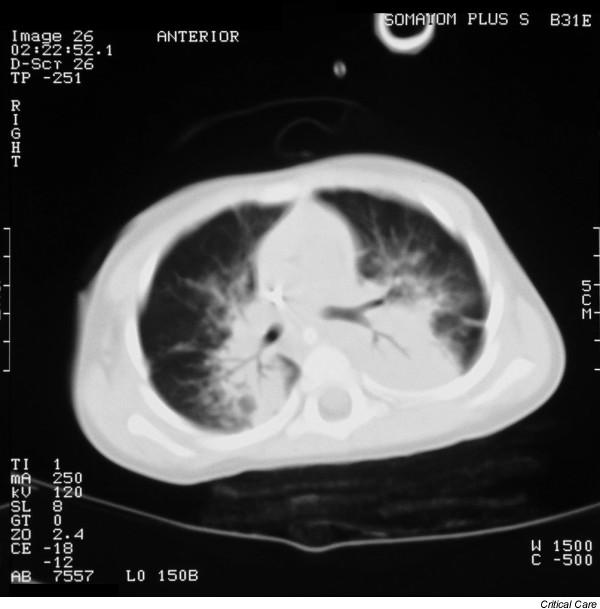
The girl: computed tomography of the thorax, done shortly after admission to hospital, showing infiltrations in the basal dorsal thorax following aspiration.

**Figure 3:**
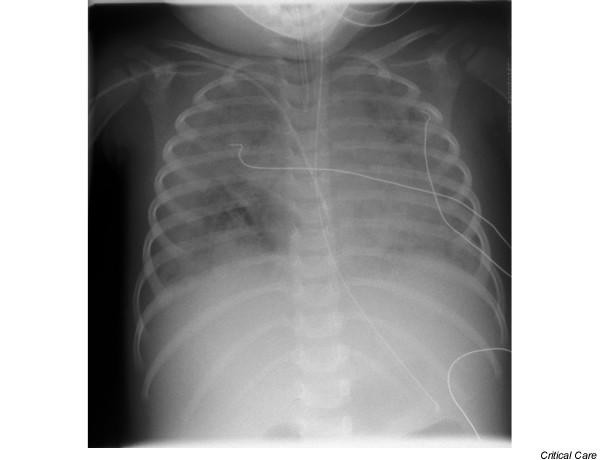
The girl: chest X-ray film, done after admission to hospital, showing increasing bilateral infiltrations of the lung, done after admission.

Under pressure controlled ventilation the oxygenation index improved initially and the inspiratory oxygen fraction could be reduced to 0.3 over the first 48 hours after the accident. However, 72 hours after the accident oxygenation deteriorated. The initial CT of the thorax had shown infiltrations in the basal dorsal thorax after aspiration (Fig. 2). The following chest X-ray films revealed increasing bilateral infiltrations of the lung (Fig. 3). After 3 days in the ICU, sepsis with multiple organ failure developed (acute respiratory dysfunction syndrome [ARDS] with an oxygenation index of 109 torr, circulatory failure requiring catecholamines, liver dysfunction with increased transferases and reduced prothrombin time, and disseminated intravascular coagulopathy). Substitution of blood products was necessary. Acute renal failure did not develop. Antibiotic treatment was started (ceftazidime for *Pseudomonas aerguinosa *in the tracheal aspirate, vancomycin for *Enterococcus faecium *at the central venous catheter tip).

Under differentiated pressure controlled ventilation, oxygenation did not improve. Because it was unclear at this time whether extracorporeal membrane oxygenation would be required, 5 days after the accident the child was transferred by helicopter to another university hospital because of limited capacity at our hospital. Under high-frequency oscillatory ventilation and nitric oxide inhalation, oxygenation improved and extracorporeal membrane oxygenation was not necessary. Conventional pressure controlled ventilation could be restored 7 days after the accident, and at the same time the multiple organ failure improved. Sedation was reduced and the girl was extubated 11 days after the accident, with no neurological deficit. Twenty-three days after the accident she was transferred to the community hospital where her brother was initially hospitalized, and she was discharged 1 day later completely restored to health.

### The boy

The brother was transported to a community hospital. Admission parameters are presented in Table 1. Haemodynamic stability was achieved 150 min after admission to the hospital under dopamine and dobutamine therapy. The pupils were slightly dilated with reaction to light and the corneal reflex was present. He was rewarmed and normothermia was achieved 5 hours after admission. Continuous catecholamine therapy was stopped 4 days after the accident. The boy was sedated with fentanyl and midazolam, and ventilated to achieve normocapnia using a pressure-controlled mode. With improvement in oxygenation, he was extubated 6 days after the accident. The initial chest X-ray films showed bilateral infiltrations of the lung as a sign of aspiration pneumonia, which improved within the next few days. Liver and kidney function remained normal.

After the end of sedation, an apallic syndrome with extension posturing developed. The initial cranial CT obtained 36 hours after admission was normal, and fundoscopy did not show signs of papillary congestion. A cranial CT obtained 32 days after the accident showed marked expansion of the internal and external cerebral fluid interspaces with marked cerebral atrophy. At discharge from hospital, 41 days after the accident, the little boy remained in an apallic state, with flexion and extension posturing.

## Discussion

We present a case of twin toddlers with different neurological outcomes after near drowning with severe hypothermia and protracted cardiac arrest. Hypothermia at the scene has yielded controversial results with respect to cerebral protection. Factors such as time to achieve hypothermia (e.g. water temperature), the degree of hypothermia, the time of submersion, and other effects such as cardiocirculatory depression or arrest have various influences on the cerebral protection conferred [3,8]. Some of these factors are unclear in this case report. The two institutional approaches to management of the twins were optimal because both hospitals have paediatric departments with paediatric ICUs. In addition, the community hospital is a training hospital and part of the university hospital.

Lavelle and Shaw [8] described three patients with body temperature under 28°C on arrival at the emergency department. All three patients had a good neurological outcome, but they fell into icy water. The use of prolonged or induced mild hypothermia for cerebral protection after near drowning has yielded controversial results [4,8]. Bohn and coworkers [6] reported on 40 children aged under 15 years who suffered severe near drowning accidents with submersion time longer than 5 min and need for CPR. Twenty-four children were treated with hypothermia (30–33°C) for 24–36 hours, and 14 survived but three of these children had permanent neurological damage. Sixteen children were kept normothermic, and 13 survived but four had permanent neurological damage. Nussbaum and Maggi [10] investigated 31 children aged under 6 years who had undergone near drowning and were in a flaccid state of coma. All children were treated with hypothermia (32–34°C) for 48 hours (half of them received additional barbiturate therapy). Twelve children recovered completely, 12 children had brain damage and seven died.

Two recently published studies, conducted in patients who had suffered out-of-hospital cardiac arrest, compared induced mild hypothermia for 12–24 hours with normothermic management [7,9]; they found that a significantly greater percentage of patients in the groups treated with mild hypothermia had good neurological outcomes. In patients affected by brain injury with a Glasgow Coma Scale score from 3 to 8, induced mild hypothermia for 24–48 hours yielded controversial findings [11,12]. In these patients hypothermia on admission correlated with poor outcome, suggesting that spontaneous hypothermia may be a result of major brain injury [11].

In the present case report, hypothermia on the scene and on admission was probably the result of external factors such as water and air temperature and the children's age, suggesting cerebral protection from hypothermia. Up until the arrival of the twins at hospital, the treatment was identical. The boy was passively warmed to achieve normothermia, and the girl underwent prolonged (72 hours) mild hypothermia (32–34°C). The different neurological outcomes could have been influenced by these different treatments. However, some factors remain uncertain. For example, was the boy the first to go into the water, with resulting longer submersion and hypoxaemia times? How effective was bystander CPR in the two children? Was the time to achieve hypothermia the same in both children? Excluding bystander CPR, the remaining factors are considered strong predictors of outcome after near drowning [1,3,8]. The girl developed ARDS and septic shock, whereas the boy recovered from aspiration pneumonia without further complications.

There is concern that prolonged mild hypothermia has adverse effects on cardiac and lung function, coagulation and the immune system [3,5,7]. In a series of 41 patients with submersion injury (temperature on admission >32°C, no induced mild hypothermia), 32% developed pneumonia and one person ARDS [8]. Significantly higher infection rates, predominantly pneumonia, were described in patients treated with induced mild hypothermia as compared with patients treated under normothermic conditions [5,13,14]. However, other investigations evaluating patients following out-of-hospital CPR and with brain damage did not identify any differences in the incidence of infection between normothermic and hypothermic groups treated. just for 12–24 hours [7,9,15]. It seems posssible that the duration of mild hypothermia has an impact in the incidence of infection and sepsis. Among the 41 normothermic patients described by Lavelle and Shaw [8], after submersion 14% developed sepsis. In experimental animal models it was shown that hypothermia under 29°C leads to a reduced neutrophil response to endotoxin [16]. Leukocytopenia has been described to be significantly more frequent in patients with induced mild hypothermia [13,14].

The girl was highly catecholamine dependent in the first 7 days after the accident. It has been reported that, in patients with mild hypothermia, significantly higher doses of catecholamines are required in comparison with normothermic patients after acute brain injury [11]. Vasopressor requirements have been described as having a significant impact on outcome [2]. The rate of other organ dysfunctions (liver, kidney) has also been found to be significantly higher in patients under induced mild hypothermia. The girl also developed transient liver dysfunction. Together with sepsis syndrome, coagulopathy developed. Disturbances of this system with resultant bleeding complications are known to occur during therapy with mild hypothermia [5,13,14,17].

## Conclusion

Although the twin toddlers experienced a near drowning accident together, the outcomes in terms of neurological status and postinjury complications were completely different. One of the factors that possibly influenced the different postinjury courses might have been prolonged mild hypothermia.

## Key messages

• 		Two twin toddlers suffered hypothermic near drowning with protracted cardiac arrest and aspiration.

• 		The girl was treated with mild hypothermia and developed acute respiratory dysfunction syndrome and sepsis, but recovered without neurological deficits.

• 		The boy was treated under normothermic conditions and developed an apallic syndrome.

• 		One of the factors that possibly influenced the different postinjury course might have been prolonged mild hypothermia.

## Competing interests

None declared.

## Abbreviations

ARDS = acute respiratory dysfunction syndrome; CPR = cardiopulmonary resuscitation; CT = computed tomography; ICU = intensive care unit.

## Figures and Tables

**Table 1:** Parameters at the scene and on admission in the twins

Site/parameter	Girl	Boy
At the scene		
Time to bystander CPR (min)	> 10	> 10
Pulseless bradycardia under CPR	Yes	Yes
On admission		
Pupils dilated, nonreactive to light	Yes	Yes
Corneal reflex	Negative	Negative
Temperature on admission (°C)	<28	<27
pH on admission	6.63	7.00
Arterial CO_2 _tension (torr)	38	36
BE on admission (mmol/l)	-27	-17
Oxygenation index (torr)	75	65
CPR time (min)	180	120

BE, base excess; CPR, cardiopulmonary resuscitation.
